# MicroRNA-Mediated Host-Pathogen Interactions Between *Bombyx mori* and Viruses

**DOI:** 10.3389/fphys.2021.672205

**Published:** 2021-05-07

**Authors:** Mian Muhammad Awais, Muhammad Shakeel, Jingchen Sun

**Affiliations:** ^1^Guangdong Provincial Key Laboratory of Agro-animal Genomics and Molecular Breeding and Sub-Tropical Sericulture and Mulberry Resources Protection and Safety Engineering Research Center, College of Animal Science, South China Agricultural University, Guangzhou, China; ^2^Laboratory of Bio-Pesticide Innovation and Application of Guandong Province, College of Plant Protection, South China Agricultural University, Guangzhou, China

**Keywords:** miRNA, BmNPV, *Bombyx mori*, host-pathogen interaction, viruses

## Abstract

MicroRNAs (miRNAs), small non-coding RNAs of about 22 nucleotides, have been reported to regulate gene expression at the posttranscriptional level and are involved in several biological processes such as immunity, development, metabolism, and host-pathogen interactions. Apart from miRNAs encoded by the host, miRNAs produced by pathogens also regulate host genes to facilitate virus replication and evasion of the host defense responses. In recent years, accumulated studies suggest that viral infections alter the host miRNAs expression profile, and both cellular and viral miRNAs may play vital roles in host-pathogen interactions. *Bombyx mori*, one of the critical lepidopteran model species, is an economically important insect for silk production. The mechanism of interaction between *B. mori* and its pathogens and their regulation by miRNAs has been extensively studied. Therefore, in this review, we aim to highlight the recent information and understanding of the virus-encoding miRNAs and their functions in modulating viral and host (*B. mori*) genes. Additionally, the response of *B. mori* derived miRNAs to viral infection is also discussed. A detailed critical view about miRNAs’ regulatory roles in *B. mori*-virus interactions will help us understand molecular networks and develop a sustainable antiviral strategy.

## Introduction

The understanding of gene expression guided by regulatory RNA molecules is not limited to the past 20 years. According to Britton and Davidson, genes might be turned on and off by activator RNA molecules based on Watson-Crick base pairing to the sites located within genes. Later, the idea was abandoned with the discovery of transcription factors (Britten and Davidson, 1969). As per our understanding, it is now clear that RNAs, especially the small RNAs (sRNA), actually regulate gene expression.

MicroRNA (MiRNA), small interfering RNAs (siRNA), and piwi-interacting RNAs (piRNA) are the three main classes of sRNAs that regulate gene expression (Farazi et al., 2008; Moazed, 2009; Czech and Hannon, 2011). These three classes are based on size and interaction with a particular protein class called the argonaute (Ago) protein family (Kim, 2008). MiRNAs are endogenous ∼22 nt RNAs that have an interaction with Ago-1 protein in insects (Kawamata et al., 2009; Miyoshi et al., 2009; Okamura et al., 2009; Ghildiyal et al., 2010). siRNA with a length of 20 nt (Zografidis et al., 2015; Santos et al., 2019) interacts with Ago-2 (Czech et al., 2009) and piRNAs of 24–31 nt with the Piwi-subfamily of Ago proteins (Siomi et al., 2011).

Two transcripts, the 22 nt lin-4 s (small) and the 61 nt lin-4 L (large) originating from the lin-4 locus, led to the discovery of the first miRNA in *Caenorhabditis elegans* (Lee et al., 1993; Wightman et al., 1993). These small transcripts were found to contain sequences complementary to the 3' untranslated region (UTR) of lin-14 mRNA, indicating that these transcripts regulate the lin-14 translation *via* some unique antisense mechanism. In 2000, after 7 years of the first miRNA discovery, the next miRNA was discovered in *C. elegans*, indicating that the 21 nt let-7 temporally regulates lin-41 by binding to the target sites within its 3' UTR (Reinhart et al., 2000).

The discovery of lin-4 and let-7 added a new dimension to our understanding of complex gene regulatory networks, and since their discovery, thousands of putative miRNAs have been identified in various organisms. MiRNAs encoded by host cells or by a viral genome are involved in the interaction between the host and the pathogen, opening new research windows in insect microbe interaction. In this review article, miRNA’s role in insect host-pathogen crosstalk, especially in *Bombyx mori*, with examples from viruses and the host with the availability of the most recent literature, will be discussed.

## miRNA Biogenesis

**Figure 1:**
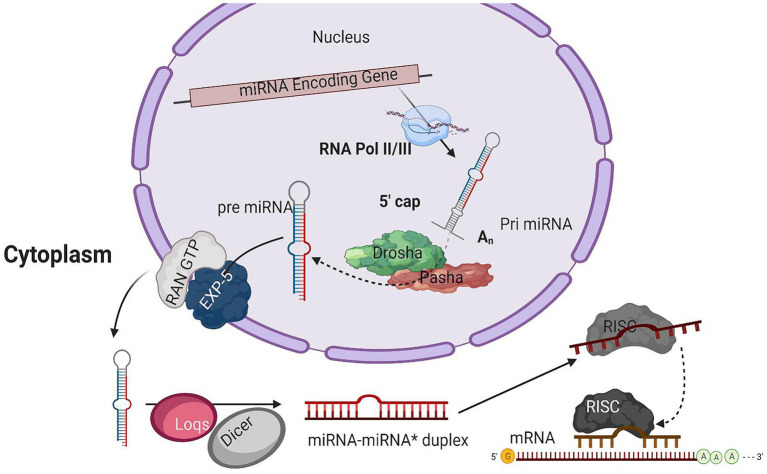
A typical model for miRNA biogenesis. RNA polymerase II(RNA pol II), transcribing miRNA gene into a hair-loop structure known as the pri-miRNA. The Drosha/Pasha microprocessor complex process pri-miRNA to pre-miRNA in the nucleus. The pre-miRNA is exported into the cytoplasm in a RanGTP/Exp-5 dependent manner. The pre-miRNA is processed by Dicer-1 (Dcr-1) and Loquacious (Loqs) to form the miRNA-miRNA* duplex within the cytoplasm. The duplex strands are then sorted, and the miRNA strand is loaded into the RISC complex that typically includes Argonaut 1 (Ago-1).

Several processing steps are involved in miRNA biogenesis, including transcription of the miRNA, loading and assembly into the RNA-induced silencing complex (RISC), and miRNA maturation. As most of the short RNAs are transcribed by polymerase III (RNA pol III), it was thought that the transcriptions of the majority of miRNAs loci are also mediated by the RNA pol III. It is also suggested that RNA pol III may mediate the transcription of miRNAs positioned within repetitive sequences (Borchert et al., 2006). On the other hand, the structure of genes encoding the miRNAs and direct experimental results indicate that RNA pol II is the primary RNA polymerase that initiates the transcription of miRNAs loci in animals. In several primary miRNA transcripts, the presence of traditional 5' 7-methyl guanosine caps and 3' polyadenylation and their sensitivity to α-amanitin having inhabiting interaction with RNA pol II suggest the class-II nature of genes having miRNA loci (Lee et al., 2004; Figure 1). All these results confirmed that RNA Poll II is involved in the transcription of miRNAs.

### The Microprocessor Complex Fine-Tunes the pri-miRNA

The initial product of RNA Poll II transcriptions is primary miRNA (pri-miRNA) transcripts. These pri-miRNAs have one or more hairpin loop structures, and their length ranges into several hundred kilobases. The microprocessor complex processes these long transcripts (pri-miRNAs) into 70 nt precursor miRNAs (pre-miRNA) in the nucleus (Conrad et al., 2014). Drosha, an RNase III enzyme along with its double-stranded RNA (dsRNA) binding partner, Pasha, constitutes the microprocessor complex (Lee et al., 2003; Denli et al., 2004; Gregory et al., 2004; Han, 2004; Landthaler et al., 2004; Kawai and Amano, 2012; Burke et al., 2014; Nguyen et al., 2015; Kim et al., 2016a). The stem structure of pri-miRNA with 30 bp has a terminal loop, and flanking segments are processed by the microprocessor complex after recognition. The dsRNA binding partner protein Pasha recognizes substrate pri-miRNA, plays an important part in anchoring to the flanking single-stranded RNA (ssRNA) and dsRNA stem junction, and locating the position 11 bp into the stem, where Dorsha is loaded to cleave the pri-mRNA (Lee et al., 2003; Denli et al., 2004). For further processing, the end product of Drosha-Pasha processing, a 70 nt pre-miRNA, is exported into the cytoplasm by Exportin-5 (Exp-5; Kim et al., 2016). The pre-miRNA generated by Drosha’s action on pri-miRNA has a 2 nt 3' overhang, critical for pre-miRNA export. Exp-5, a dsRNA-binding receptor depending on ran guanosine triphosphate (RanGTP), starts the nuclear export of pre-miRNAs by identifying the 2 nt 3' overhang structure of pre-miRNA in the nucleus (Bohnsack, 2004). The Exp-5/pre-miRNA complex migrates through the nuclear pore complexes into the cytoplasm. In the cytoplasm, RanGTP is hydrolyzed to RanGDP, which results in the release of pre-miRNA from the Exp-5/pre-miRNA complex. The pre-miRNAs are protected from digestion by nucleases of Exp-5, which is also the nuclear export factor of pre-miRNAs (Yi, 2003; Lund, 2004).

### Pre-miRNA Processing by Dicer

An RNase III enzyme, Dicer-1 (Dcr-1), slices the terminal loop structure of pre-miRNA, releasing a ~22 nt miRNA-miRNA* duplex in the cytoplasm (Hutvagner, 2001; Koscianska et al., 2011; Feng et al., 2012; Gurtan et al., 2012; Bogerd et al., 2014). Dicer, a core enzyme in the RNAi pathway, was first identified in *Drosophila* (Bernstein et al., 2001). Two Dicer enzymes, Dcr-1 and Dcr-2, are encoded by the *B. mori* genome having the specialized function in two crucial pathways, i.e., miRNA and siRNA pathways (Ponnuvel et al., 2007; Kolliopoulou and Swevers, 2013). A pre-miRNA processing complex is formed by the interaction of Dcr-1 with Loquacious (Loqs) (Saito et al., 2005). The pre-miRNA is accumulated, and mature miRNAs are reduced when RNAi depletes the Loqs; depletion of Dcr-1 also results in similar effects (Jiang, 2005).

### miRNA Strand Selection and Argonaute Loading

From mature miRNA-miRNA* duplex resulted from pre-miRNAs processing by Dcr-1, a strand from the duplex is loaded into the RISC. RNA-binding proteins guided by sRNA from the argonaute family are the fundamental components of the RISC. Four discrete ago proteins encoded by the *B. mori* genome are classified into two sub-clades: Ago and Piwi (Wang et al., 2013). Subgroup ago includes the ago-1 and ago-2 proteins that bind with miRNAs and siRNAs, respectively (Hammond, 2001). The mature miRNA guide strand is loaded into ago-1, while the miRNA* strand is degraded (Kawamata et al., 2009). The miRNA-Ago complex is ready to perform its action on target sequences.

## miRNAs Modulating Host-Pathogen Interaction

The role of miRNAs in insect development has been studied extensively. A lot has also been studied in recent years related to miRNAs’ functional role in infection establishment as these modulate the host-pathogen interactions. For instance, quite a few reports describe viral and cellular miRNAs’ involvement in infection propagation by limiting viral replication (see below). The expression profile of a large number of miRNAs has been shown to alter upon the pathogen’s invasion. The modulation of host and pathogens genes is also reported by the miRNAs encoded by the host and pathogens. Below, we will examine the manipulation of genes from different miRNAs sources, which intern mediate the host-pathogen interaction.

### Impact of Viral Infection on the Host miRNA Profile

**Figure 2:**
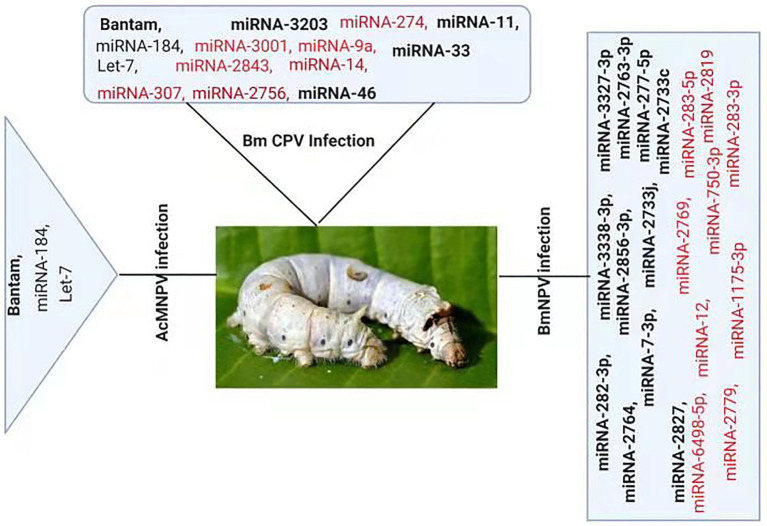
Differential Expression of host miRNA upon viral invasion: Regulation of miRNAs upon BmNPV, BmCPV, and AcMNPV. Red color represents down-regulated, while bold black represents up-regulated miRNAs after the viral attack.

Several studies have reported change in cellular miRNAs expression levels in insects in response to pathogen infection based on deep sequencing or microarray analysis (Figure 2). For example, Karamipour investigated the difference in the expression profile of cellular miRNA-184 and let-7 following *Autographa californica* nucleopolyhedrovirus (AcMNPV) infection of Sf-9 cells. Findings suggested that following AcMNPV infection, there was a difference in the expression of cellular miRNAs at the post-infection times. The expression profile of the core components of miRNA’s biogenesis pathway, Dcr1, Ago1, and Exp5, was upregulated at 16 h post-infection (hpi) following AcMNPV infection. Though, in response to virus infection, Ran’s expression was decreased (Karamipour et al., 2019). An abundant miRNA, Bantam, accounts for more than 5% of the total miRNA of Sf9 cells. Sequenced data suggested that the bantam level was increased late in AcMNPV infection and confirmed with real-time quantitative PCR in infected Sf9 cells and *Spodoptera litura* larvae (Shi et al., 2016).

At 24 h pi, there was an increase of 2.5-fold in bantam level in uninfected cells, and a high level of bantam was also present late in the infection. The bantam level was also about 1.5-fold more elevated than the control in AcMNPV-infected *S. litura* larvae, indicating that bantam has a role in the growth and modulates the insect-baculovirus interaction as well (Shi et al., 2016).

Deep sequencing of *Bombyx mori* cytoplasmic polyhedrosis virus (BmCPV) infected midgut of silkworm suggests that the expression profile of host miRNAs were altered after 72 and 96 h of viral inoculation. In the RNA libraries constructed from the BmCPV-infected midgut of silkworm and the control midgut, a total of 316 known miRNAs (including miRNA*) and 90 novel miRNAs were identified. A significant difference in the expression of 58 miRNAs was observed between the infected and the normal midgut (Wu et al., 2013). Two small RNA libraries were constructed from *Bombyx mori* nuclear polyhedrosis virus (BmNPV) infected larvae and the control larvae. Solexa sequencing results revealed significant downregulation of miR-2819 in infected larvae (Wu et al., 2019). Results showed that miR-2819 has a relatively high abundance in several tissues, including Malpighian tubules, fat body, hemolymph, silk gland, and midgut. The abundance of miR-2819 of infected cells was compared with that of control cells at different time points. The results showed that during the 6–12 hpi, the expression level of miR-2819 was increased, while during 24–72 hpi, the expression level of miR-2819 was significantly declined (Wu et al., 2019).

The knowledge about the modulation by miRNAs in the establishment of infection is increasing as more of these studies are emerging. New dimensions are now discovered how the complex phenomena of host-pathogens occurred upon pathogens invasion and how the host responds to these invading pathogens.

### Host miRNA’s Impact on Viral Infection

Cellular miRNAs may directly target viral genes and impact the propagation and establishment of infection (Table 1). Apart from regulating cellular processes, host miRNAs also have a crucial role in defense against pathogen attack, thus helping in infection propagation (Skalsky and Cullen, 2010; Asgari, 2011; Hussain and Asgari, 2014). miRNA also addresses the biotic stresses like protein-coding genes (Ghosh et al., 2009; Croston et al., 2018). A large number of miRNAs have a differential expression upon pathogen attack (Ghosh et al., 2009; Maudet et al., 2014; Croston et al., 2018). This differential expression of miRNAs indicated the critical role they play in host-pathogen interactions. For instance, with various putative binding sites, *B. mori* miR-8 was defined as an antiviral miRNA. The putative binding sites were on the BmNPV immediate-early gene (ie-1) mRNA and other vital genes of BmNPV. On blocking of Bmo-miR-8, a 3-fold increase in the ie-1 transcript level and an 8-fold increase in BmNPV accumulation in fat body tissues of infected larvae was observed, indicating a significant increase in the virus load in the infected *B. mori* larvae (Singh et al., 2012). Similarly, the role of bantam on viral infection was studied. The results indicated that cellular miRNA bantam in Sf9 cells plays an essential role in insect growth and baculovirus-insect interaction. The expression of the most affected viral genes lef8, gp41, and p10 was increased by 8, 10, and 40 times after applying bantam inhibitor or mimic in Sf9 cells. In infected *Spodoptera exigua*, larval mortality increased from 47% without antago-miR (bantam inhibitor) to 80% with it (Shi et al., 2016). In another study, it was demonstrated that miR-278-3p plays a vital role in BmCPV replication. Insulin-related peptide-binding protein 2 (IBP2) gene, induced by BmCPV infection, having a vital role in *B. mori* immune response, was identified as one of the targets of miR-278-3p by using a luciferase reporter assay. Over-expression of miR-278-3p negatively regulates the expression of IBP2 in silkworm larvae and positively regulates the mRNA transcript level of BmCPV, and confirms that the miR-278-3p is important for viral infection establishment (Wu et al., 2016). *Bombyx mori* miR-2,819 effectively downregulates the immediate early gene ie-1 required for viral replication, affecting the viral infection. miR-2,819 also affects other gene’s expression, i.e., polyhedrin and VP39 of BmNPV (Wu et al., 2019).

**Table 1:** Host miRNAs targeting viral genes.

Host miRNAs	Functional target in the viral genome	Function	References
miR-8	BmNPV immediate-early gene (ie-1)	Replication	Singh et al., 2012
bantam	lef8, gp41, and p10		Shi et al., 2016
miR-278-3p	Insulin-related peptide-binding protein 2 (IBP2)	*B.mori* immune response	Wu et al., 2016
miR-274-3p	BmCPV nonstructural protein 5 (NS5) and p10	Viral replication	Wu et al., 2017
miR-2,819	BmNPV immediate-early gene (ie-1)	Replication	Wu et al., 2019
miR-390	BmNPV-cg30	Occlusion bodies formation	Kang et al., 2018

Accumulation of 20-hydroxyecdysone (20E) in the late phase of larval instar results in the initiation of molting and metamorphosis (Riddiford et al., 2003). MiRNA bantam affects baculovirus-host interaction by regulating 20E. Reduction in bantam level results in an increased level of 20E, resulting in high mortality in virus-infected *S. exigua* (Ran et al., 2018). Bmo-miR-390 effectively downregulates the expression of BmNPV-cg30 in BmNPV-infected BmN cells. Cg30 gene of BmNPV is required to replicate BmNPV as a deletion or mutation in this gene results in decreased occlusion bodies (OB) production and reduction in toxicity to the silkworm larvae (Ishihara et al., 2013; Kang et al., 2018).

The impact of miR-274-3p on the BmCPV replication in the silkworm larvae infected with the BmCPV was investigated. The experimental techniques, including bioinformatics analysis, identified BmCPV Nonstructural protein 5 (NS5) as the potential target of miR-274-3p. qRT-PCR and Western blotting results revealed that the level of NS5 was reduced significantly by miR-274-3p inhibitors, while the polyhedrin gene expression was increased after the application of miR-274-3p inhibitors. The inhibition of miR-274-3p facilitates BmCPV replication by upregulating BmCPV NS5 gene expression (Wu et al., 2017). The authors demonstrated that mature artificial miRNAs (amiRNAs) expressed successfully target the viral lef-11 gene. Mature amiRNAs efficiently inhibited the BmNPV proliferation by silencing the target gene. The overexpression of mature amiRNAs may induce acute cellular toxicity (Zhang et al., 2014). Next-generation sequencing (NGS) showed that 167 genes were upregulated and 141 genes were downregulated in larval instars of *B. mori* following pathogenic infection. Several genes with a role in *B. mori* immune response against BmCPV were identified. The 2-fold upregulation of the core RNAi genes Ago-2 and Dcr-2 was observed during pathogenic infection (Kolliopoulou et al., 2015). Reports revealed that exo-RNAi is operative in the silkworm *B. mori* against pathogenic infection of BmCPV, which is characterized by a segmented dsRNA genome (Zografidis et al., 2015).

### Viral miRNA Can Target Viral Own Genes

MicroRNAs carry out their functions through their targets, and for viral miRNAs, their targets can be the host genes or the viral genes. MiRNAs can bind to the 3' or 5' UTR of the target mRNA, and they also can bind to coding sequences to manipulate target gene expression (Table 2). For instance, bmnpv-miR-3 miRNA encoded by BmNPV modulates the expression of DNA binding protein (P6.9), vital for the late stage of viral infection in the host, *B. mori*. The downregulation of BmNPV late genes helps BmNPV escape the host’s early immune response (Singh et al., 2014). The viral miRNAs are mainly involved in the active regulation of viral genes. It may reduce the viral DNA load and the number of infectious budded virions (BVs) to avoid host surveillance and prolong residence duration inside the host cell.

**Table 2:** Viral micro RNA can target viral own genes.

Viral miRNA	Target genes	Function	References
BmNPV-miR-3	P6.9-gene	Viral infection establishment	Singh et al., 2014
AcMNPV-miR-1	Ac-94-gene	Viral DNA replication	Zhu et al., 2016
AcMNPV-miR-3	Ac101-gene	BV and ODV production	Jiao et al., 2019
AcMNPV-miR-1	Ac-95(down regulation) & Ac-18 (up Regulation)	DNA helicase, replication related	Wang et al., 2020a
AcMNPV-miR-2	lef-6, lef-11, orf-49, and orf-63	DNA replication, late gene transcription	Wang et al., 2020a
AcMNPV-miR-3	Ac23, ac25, ac86, and ac98	Polynucleotide inase/ligase and fusion protein	Wang et al., 2020a

AcMNPV-miR-1 is an AcMNPV encoded miRNA. The ac-94 gene involved in producing infectious BVs was downregulated by AcMNPV-miR-1, resulting in the decreased production of BVs and enhancing the formation of occlusion-derived virions (ODVs). The overexpression of AcMNPV-miR-1 reduces the budded virus’s infectivity, affecting viral DNA replication and accelerating ODVs formation. AcMNPV-miR-1 moderately downregulated ac95 and upregulated ac18. These findings suggest that AcMNPV-miR-1 restrains virus infection of cells but facilitates virus infection of larvae (Zhu et al., 2016; Wang et al., 2020a). The most distinct downregulation for AcMNPV-miR-2 was observed for lef-6, with lef-11, orf-49, and orf-63 also showing apparent downregulation, while orf-38 did not exhibit significant regulation (Wang et al., 2020a). AcMNPV-miR-3 plays a regulatory role in BV and ODV production. AcMNPV-miR-3 is located on the opposite strand of the viral gene ac101 coding sequence in the AcMNPV genome and detected at 6 hpi. and reaches a maximum level of around 12 hpi. in AcMNPV-infected Sf9 cells. AcMNPV-miR-3 downregulates ac101 through a siRNA-like cleavage mode. Ac101 is a core gene required for BV and ODV production, viral infectivity, and virus-induced nuclear actin polymerization. Dual-luciferase reporter assay revealed a slight downregulation with ac23, ac25, ac86, and ac98 target sites (Jiao et al., 2019; Wang et al., 2020a).

The cellular state from susceptible to resistant or vice versa may be remodeled upon abnormal-expression of cellular miRNAs due to the abnormal expression of their target genes (Ghosh et al., 2009). A huge amount of literature is available describing miRNAs’ identification from insect hosts, but the exact role in host-pathogen interactions is still limited to a few reports (Ylla et al., 2016; Kozomara et al., 2019). Moreover, these studies mainly focus on miRNA alterations due to the viral attack in insects (Tang et al., 2019).

### Viral miRNAs Exploit the Host Machinery

Recent investigations have revealed that viral miRNAs might target and regulate host genes to establish infection and successfully replicate. However, the information is minimal concerning insect viruses. The miRNAs’ impact might be *via* regulation of a specific gene leading to modifying a particular pathway or might cause a global reduction in the host miRNA depending on the target genes (Table 3). For instance, a BmNPV encoded miRNA (bmnpv-miR-1) has been shown to downregulate the host GTP-binding nuclear protein Ran’s expression, thereby inhibiting small RNA transport from the nucleus into the cytoplasm to ensure their active proliferation (Singh et al., 2012). Reports indicated that an insect double-stranded RNA virus (BmCPV) might generate miRNAs, and these miRNAs play an essential role in host viral interaction. BmCPV-miR-3 and BmCPV-miR-5 regulate host target genes and manipulate viral replication and proliferation (Pan et al., 2017) AcMNPV encoded miRNA AcMNPV-miR-4 interfere with the host cell cycle, cytokine secretion, exocytosis, and membrane fusion as it targets the ALG2 and ROP genes. ALG-2 is involved in apoptosis initiation. Rop is highly expressed in the nervous system and in specialized tissues where intensive exocytic/endocytic cycles occur, providing evidence that it impacts viral infection and propagation (Jiao et al., 2019). A BmCPV-derived miRNA (BmCPV-miR-1) effectively up-regulates the expression of the *B. mori* inhibitor of apoptosis protein (BmIAP) gene. It inhibits cell apoptosis mechanisms, hence favoring better replication of the virus and helps in viral infection establishment (Guo et al., 2020).

**Table 3:** Viral mRNAs exploit the host machinery.

Viral mRNAs	Targets gene in host	Function	References
BmNPV-miR-1	GTP-binding nuclear protein Ran encoding gene	Inhibits transport from nucleus to cytoplasm	Singh et al., 2012
BmNPV-miR-415	TOR2	Metabolism and development	Cao et al., 2017
BmCPV-miR-3	Purine nucleoside phosphorylase encoding gene	Cell proliferation inhibition	Pan et al., 2017
BmCPV-miR-1	BmIAP gene	Inhibits cell apoptosis	Guo et al., 2020
AcMNPV-miR-4	ALG2 and ROP genes	Apoptosis initiation blocked	Jiao et al., 2019

E66 a structural protein of the ODV encoded by ORF37 of the BmNPV genome. It also encodes a miRNA BmNPV-miR-415, which produces bmo-miR-5738 when introduced into the host cells. Bmo-miR-5738 upregulates the expression of TOR2 through its 3' UTR. TOR2, as a member of the phosphatidylinositol kinase-related kinase family, plays a critical role in metabolism, development, growth, and survival at the cellular and organism levels. Recently it has been revealed that TORE2 also upregulates the molting hormone 20E (Cao et al., 2017). Apoptosis in Sf9 cells due to the inhibition of miR-14 indicates that it is essential for constitutive cell survival. When mimics of miR-14 precursor molecules were applied, they partially inhibited the cell death induced by actinomycin-D (Act-D). MiR-14 might have inhibitory interactions with caspases, as it functions downstream of mitochondrial cytochrome and hence averting Act-D-induced apoptosis (Kumarswamy and Chandna, 2010). Similar exploitation of host-pathogen interaction by miRNAs was also observed in other diamondback moths. Certain miRNAs are also produced by *Cotesia vestalis*, (a parasitoid of *Plutella xylostella*) and bracovirus (CvBV). The parasitized host revealed that most of the miRNAs that arrest the host’s growth were of *C. vestalis* origin. In contrast, the expression profile of the miRNAs encoded by CvBV was 100-fold increased in parasitized hosts than non-parasitized ones. miRNAs arrest the growth of the host by modulation of the host ecdysone receptor (EcR; Wang et al., 2018).

## Interaction of miRNAs with Other Coding and Non-Coding Transcripts

With the invention of modern techniques and a better understanding of genomic approaches, it is clear that miRNAs can have regulatory interaction with other transcripts. This complex interplay of miRNAs with these transcripts mediates the regulatory role of the different transcripts. A recent report suggests that differentially expressed long non-coding RNAs interacting with the target genes and miRNAs participate in the host response to the viral infection (BmNPV) by targeting the genes enriched ubiquitin-mediated proteolysis endocytosis and lysosome pathways in *B. mori* (Zhang et al., 2020).

**Figure 3:**
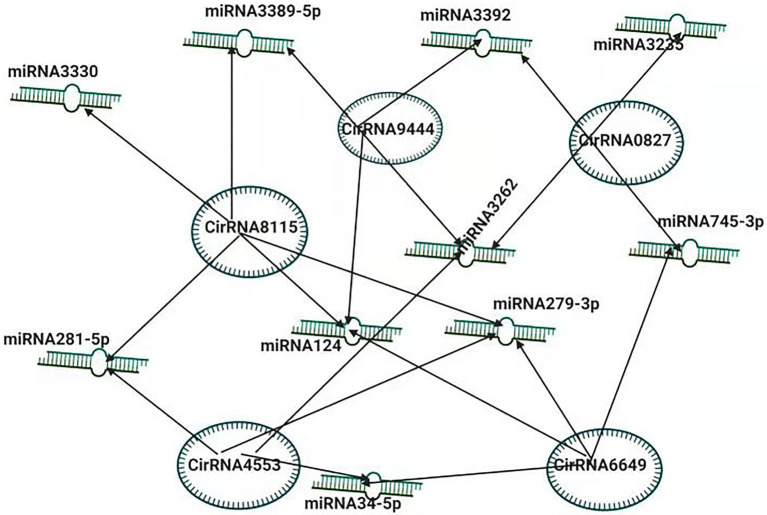
Inter-Play between miRNA and non-coding circRNAs. The circRNA-miRNA co-expression network in silkworm. circRNAs that sponged more than four miRNAs and modulating the vital processes are shown.

To better understand the pathogenesis of BmCPV, the role of circular RNAs (circRNAs) acting as positive regulators in BmCPV infections was investigated. The researchers constructed the circRNA-miRNA interaction networks based on correlation analysis between the differentially expressed circRNAs and their miRNA binding sites. The constructed complex explained an abundant interaction between miRNAs and circ RNAs, indicating the possible role of miRNAs in association with circular RNAs (Hu et al., 2018). The circRNA circEgg encoded by *B. mori* histone-lysine N-methyltransferase eggless (BmEgg) gene positively regulates histone deacetylase (HDAC) Rpd3 (BmHDAC Rpd3) gene expression by sponging the miRNA bmo-miR-3,391-5p (Wang et al., 2020b; Figure 3).

In one study, miRNAs’ role in host specificity was examined as miRNAs regulate the virus-host interaction. The downregulation of several genes determined the specificity. The screening of miRNA induced by AcMNPV infection combined with NGS predicts possible regulation networks indicating that these are also involved in the host specification of the virus (Chen et al., 2018).

## Conclusion

With the discovery of the first miRNA nearly 30 years ago and the invention of new techniques, we have started to understand these regulatory molecules’ biogenesis and diversity. The identification and characterization of miRNAs is a rapidly growing area of research. Continuing genome-wide efforts in insect miRNA discovery and expression profiling have revealed that conserved and species-specific miRNAs may play important roles in insect biology. Recently, the role of these miRNAs in host-pathogen interactions has been reported by several publications, with the majority of researchers concentrating on the impact of infection on host miRNA profile. Understanding the role of miRNAs and their targets as modulators of the insect-virus interaction can open avenues for using miRNAs, targets, and miRNA modulation pathways as a novel approach for managing viral infections and disrupting the epidemiological cycle of transmission. The host-viral interactions mediated by miRNAs provide us the basis for the use of miRNAs for insect control (Monsanto-Hearne and Johnson, 2018). Previously, similar techniques were employed by other groups when the introduction of the inverted repeat-containing transgene was made, which in turn results in *B. mori* resistance to baculovirus (Sahara et al., 2003; Subbaiah et al., 2013).

MicroRNA profile of the host is generally altered upon the infection, but the amount of damage depends on the host-pathogen interaction. The host miRNA profile change might be the host response to pathogen attack, which modeled the signaling pathway and immune responses or provided aid to pathogen replication and manipulation as an optimal environment. Important regulatory roles, i.e., suppression of host anti-pathogen responses, regulation of pathogen replication, are played by the miRNAs encoded by the host to avoid the host’s demise. In that context, the role of small non-coding RNAs in cross-kingdom and cell-to-cell communications in secreted forms such as exosomes opens up new windows of research. An exciting unmapped area is the involvement of miRNAs in the interaction of the gut microbiome with insects. With recent reports from mammals, it is suggested that host miRNAs might adjust the microbiota by modulating the bacterial gene transcription and hence have effects on their growth (Liu et al., 2016). It will not be surprising if similar associations exist in insects. Another remarkable aspect of miRNAs in host-pathogen interaction is their application in pest control. Zhang et al. (2014) reported the inducible and related production of amiRNAs to limit the pathogen infection in beneficial insects such as *B.mori*. In the same way, miRNAs may well be engaged to improve the effectiveness of biocontrol agents used against agricultural pests. In-depth investigations are required in this aspect. Several other publications also focused on the differential abundance of miRNAs. Experimental methodologies explaining the role of the differentially abundant miRNAs in host-pathogen interactions and insect immunity are of great importance. Several miRNAs encoded by viruses have also been identified, affecting virus replication by regulating or modulating the host genome facilitating their replication. These miRNAs modulate the host and viral transcripts during viral infection. To develop novel strategies to reduce the risk of viral infection the mechanisms of miRNA-mediated processes provide us the basis.

A given miRNA can target hundreds of different mRNAs, and on the other hand, a mRNA can be regulated by different miRNAs. This complex interplay is far from simple, and we are far from fully understanding the complex molecular mechanisms that regulate the crosstalks between baculovirus encoded miRNAs and insects. Further researches will be required to validate these scientific issues.

Additionally, the mechanisms by which cellular miRNAs are degraded after they finish their task needed to be clarified. Otherwise, they will affect cell metabolism and interfere with cellular functions. McCaskill reported RNA-mediated miRNA degradation, but it remains unclear whether the mechanism is a widespread viral strategy (McCaskill et al., 2015). Therefore, further investigations are required to answer the question.

## Author Contributions

MA, MS, and JS drafted the manuscript. MA and MS equally contributed to the development of this manuscript. MS and JS assisted to revise the manuscript. All authors read and approved the final manuscript.

### Conflict of Interest

The authors declare that the research was conducted in the absence of any commercial or financial relationships that could be construed as a potential conflict of interest.
